# Post partum anxiety and depression in peri-urban communities of Karachi, Pakistan: a quasi-experimental study

**DOI:** 10.1186/1471-2458-9-384

**Published:** 2009-10-12

**Authors:** Niloufer S Ali, Badar S Ali, Iqbal S Azam

**Affiliations:** 1 Department of Family Medicine, Aga Khan University, Stadium Road, P. O. Box 3500, Karachi 74800, Pakistan; 2 Department of Community Health Sciences, Aga Khan University, Karachi, Pakistan, Stadium Road, PO Box 3500, Karachi 74800, Pakistan

## Abstract

**Background:**

Postpartum anxiety and depression is a major public health concern because of its adverse effects on the cognitive and social development of the infant. Globally postpartum depression has been widely investigated but as anxiety is a more prominent feature of postpartum depression we assessed the prevalence of anxiety and depression and their associated factors in post partum women.

**Methods:**

A quasi-experimental study investigating the impact of postpartum anxiety and depression on child growth and development was conducted in two peri-urban, multiethnic, communities of Karachi, a mega city of Pakistan. A house to house questionnaire based survey was done by trained field workers; 420 consenting pregnant women were identified and data for socio-demographic, home environment and family relationship variables was collected between 36 weeks of pregnancy and within 10 days of childbirth. Mother's levels of anxiety and depression were assessed after one month, two months, six months and twelve months of childbirth; this was two step process: initially an indigenous, validated screening instrument Aga Khan University Anxiety and Depression Scale was used and diagnostic confirmation was done through a psychologist's interview based on DSM IV criteria. Women found to be anxious and depressed at least once out of four assessments were considered for the computation of overall prevalence of postpartum anxiety and depression as well as its risk factors. However, point prevalence's of postpartum anxiety and depression were also reported at each assessment time. Two sixty seven women could be followed for one year. Data was analyzed using SPSS. Chi-square test, simple and multiple logistic regression were used to see the association of different factors.

**Results:**

The overall prevalence of postpartum anxiety and depression was found to be 28.8 percent. Domestic violence, difficulty in breast feeding at birth and unplanned current pregnancy were found to be significantly associated with postpartum anxiety and depression.

**Conclusion:**

Domestic violence and not having the right to plan pregnancy are related to the patriarchal culture and lack of empowerment of women. The association with difficulties in breast feeding needs to be further explored in future studies

## Background

Postpartum anxiety and depression is a major public health concern as it is the leading cause of maternal morbidity. It is associated with adverse effects on the cognitive and social development of the infant [1]. In the postpartum period half to two thirds of women suffer from mood disturbances, for most symptoms are transient and relatively mild, known as postpartum blues [2] and settles spontaneously within four weeks. Therefore, postpartum depression (PPD) is typically diagnosed during 4-12 weeks after childbirth [3]. There is a critical period for the development of affective disorders from birth to the first year postpartum [4] and so depression occurring within a year after childbirth can be labeled as PPD [5].

Varying figures for prevalence of PPD have been reported from different countries from as low as 11% to as high as 42% [6-14]. Studies done in urban tertiary care settings in Pakistan, had reported figures ranging from 24% - 42% [8,13]. Whereas community based studies from rural Pakistan have reported prevalence's ranging from 28% - 36% [11,14]. A cohort study from rural Pakistan has reported persistent PPD (found depressed at all three time points in the first postnatal year) of 56% [15].

Risk factors for postpartum depression already identified are: personal history of earlier depression particularly antenatal depression, illiteracy, low socio-economic status, anxiety during pregnancy, experiencing stressful life events during pregnancy/puerperium, female infant gender, low levels of social support and poor marital relationship [9,11,13,14,16-19]. Most of these risk factors have also been demonstrated in developed countries [20].

Numerous studies have been done on prevalence and determinants of PPD in developed countries, but there is still scarcity of data in our local context which can be generalisable. Therefore, we aimed for this study to determine the prevalence and associated factors of postpartum anxiety and depression in multi-ethnic urban population. Here we have used the term postpartum anxiety and depression, as anxiety is a more prominent feature of PPD than of depression that occurs at other times in life [4,21].

## Methods

### Study design, site and duration

This was a quasi-experimental study investigating the impact of postpartum anxiety and depression on child growth and development; conducted in two peri-urban, multiethnic, communities of Karachi: Qayoomabad and Manzoor Colony, from February 2004 - December 2006. Karachi is a mega-city, an industrial hub, the largest port and the busiest airport of the country; their inhabitants represent nearly all the ethnic groups in the country and is referred to as Mini-Pakistan. Mostly immigrants who come to the city in search of a livelihood usually live in peri-urban areas because of dearth of housing within the city. Based on this reality the field sites were chosen; with the expectation that the results would probably be more generalisable to other areas of the country.

### Study participants and Sample

**Figure 1:**
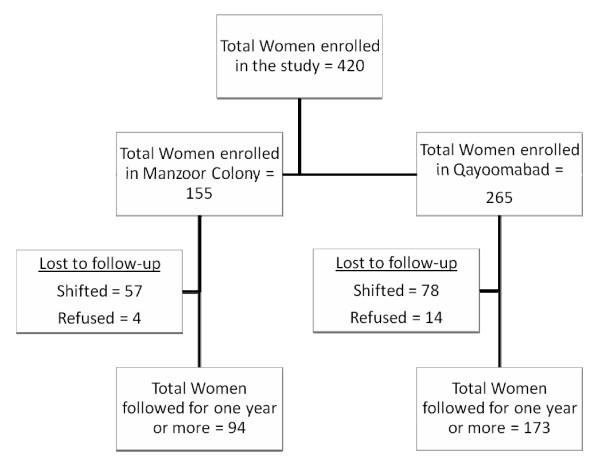
**Flow Chart Showing the Enrollment and follow up status of Women in the Study**.

All pregnant women who were living in the defined sites were identified by house to house survey and obtained consent for participation after giving a live childbirth. A total of 420 women were enrolled who had given consent out of 651 pregnant women identified, during February 2004 till December 2005. However 267 could be followed for one year (Figure 1). The overall recruitment rate was 64.5 percent (420 out of 651). It was 67.8 percent (265 out of 391) for Qayoomabad and 59.6 percent (155 out of 260) for Manzoor Colony.

### Instruments used

***a. Socio-demographic questionnaire ***consisting of: mother's age, religion, ethnicity, mother education and occupation, husband education and occupation, household monthly income (in Pakistani Rupees), ownership of house, total number of rooms, total number of household members, number of pregnancies, number of live births, number of abortions/stillbirths, number of children died, reasons for death of children, planned or unplanned current pregnancy, ever used or intention to use any contraceptive method and autonomy to use contraceptive method.

***b. Home environment/Family relationship questionnaire ***consisting of: presence or absence of stressful home environment, satisfaction with current life, any family/social support to cope with stress, decision maker of the household matters, mother ever abused physically or verbally by any family member (including during current pregnancy) and children ever abused physically or verbally by any family member.

***c. Post-natal questionnaire ***consisting of: gender of the child, date of birth, weight (in Kg.) of the child, place of birth, person conducted the delivery, any complication during child birth, and any complications in the newborn.

***d. Aga Khan University Anxiety and Depression Scale (AKUADS)***: It is a screening instrument for anxiety and depression developed indigenously from symptoms of patients suffering from anxiety and depression recorded verbatim in the local language Urdu, [22] and subsequently validated by keeping the psychiatrists interview as the gold standard. At a cut off score of 19 it has a sensitivity of 74%, specificity of 81%, a positive predictive value of 63% and a negative predictive value of 88% [23]. It is a good screening instrument to be used locally as it has a high negative predictive value, a high linguistic validity and it is easy to train local field workers in its administration, because of these attributes it has been used in several studies in Pakistan [24-27]. As anxiety and depression mostly co-exist and in fact anxiety is a more prominent feature of postpartum depression, it was decided to use AKUADS in this study. As AKUADS is a screening instrument, diagnostic confirmation was established by a clinical psychologist. Mothers who tested positive on AKUADS (score of 19 or above) and those who were marginally below the cut off score i.e. with scores of 17 to 18 were interviewed by a clinical psychologist for confirmation of diagnosis according to DSM IV criteria. This process was followed at each assessment time.

### Definition of Postpartum anxiety and depression

Any women found anxious and depressed on AKUADS scale and confirmed by a clinical psychologist's interview during 1 to 12 months after childbirth. The timings of assessment were 1 month, 2 months, 6 months and 12 months.

Women found to be anxious and depressed at least once out of four assessment times were considered for the computation of overall prevalence of postpartum anxiety and depression as well as for its risk factors. However, those women found to be anxious and depressed at a particular assessment time were considered for the computation of point prevalence of postpartum anxiety and depression.

### Selection of field workers and Training

Women residing at the study sites, able to read and write Urdu (the lingua franca) and willing to be trained and to work in the field as a paid worker were identified. They were trained in administration of the screening instrument AKUADS, and other study questionnaires about socio-demographics, home environment, family relationships and post-natal information. They were also trained for providing counseling sessions to those mothers who found to be anxious and depressed to help them to cope with anxiety and depression. Out of the 19 trained women 11 were selected as field workers based on their ability to maintain confidentiality, to communicate empathically and having permission from their families to move freely in the community. A higher number were trained to meet any dropouts from the selected field workers.

### Implementation

These field workers visited each house in both the field sites to identify pregnant women of any gestational period and obtain informed consent for participation after child birth. The expected date of delivery was calculated from the date of the last menstrual period. The field workers started weekly home visits after the 36^th ^week of pregnancy. Mother's were enrolled within 10 days of child birth and were administered the socio-demographic, home environment and family relationship questionnaires. AKUADS was administered after taking consent again, initially after 1 month, and subsequently after 2 months, 6 months, and 12 months of child birth. Efforts were made to ensure privacy as much as possible during the interviews including asking for an appropriate time when women were likely to be alone at home. In a few cases where this was absolutely impossible the sessions were conducted at the study field office. The participation was voluntary as consent was obtained more than once; before enrollment, before administering questionnaires and prior to administration of AKUADS at each assessment time and also before clinical psychologist interview. Those women who had mild to moderate depression were counseled for 8 weekly sessions by field workers and those with severe depression were referred for treatment.

### Ethics approval

The project was funded by the Aga Khan University Research Council Grant and ethical approval was obtained from the Ethics Committee of the Aga Khan University.

### Data management and analysis

Data was double entered using EpiData (version 3.02) package; 10% of the records were randomly checked to assess the quality of data entry. The data entry error rate was observed as less than 3 per 1000 fields entered. Data was analyzed using the statistical software package SPSS (version 15.0). The percentage distribution of different characteristics of mothers, children and households was generated for lost to follow and those followed up and their associations were observed using chi-square test.

Further analysis included only those who were followed up for one year. Point prevalence at 1 month, 2 months, 6 months and 12 months and overall prevalence (any time after 1 month till 1 year) of post partum anxiety and depression among mothers were computed. Overall prevalence was also computed for different characteristics of mothers, children and households.

Domestic violence was assessed using variables on mother or children who were physically, verbally or emotionally abused by any family member.

Crude odds ratios with 95 percent confidence intervals were computed for the variables of characteristics mentioned above using simple logistic regression. All variables having a p-value less than 0.25 at univariate level were considered eligible for multivariable analysis. Study area and mother's age were also included in the multivariable analysis as proxy to immigrant status and for biological importance respectively. Cramer's V was also used to assess multicollinearity between independent variables, a value of 0.8 or more was considered multicollinearity. Adjusted odds ratios with 95 percent confidence intervals were computed using multiple logistic regression.

## Results

A total of 420 women were enrolled in two peri-urban settlements of Karachi, only 63.6% (267 women) could be followed for one year, the reasons for lost to follow were shifting of residence from the sites of the study or refusal to continue after having initially agreed to do so. No significant association was observed between those who were followed for one year and those who had lost to follow except that there were fewer lost to follow in the group that owned their houses (p-value = 0.013), this does not mean that one group is socio-economically better than other as shifting residence can occur in both situations. Baseline socio-demographic characteristics of the study population are summarized in table 1, and personal characteristics in table 2.

**Table 1:** Socio-demographic Characteristics of Women in the study area (n = 420)

**Characteristics**	**Lost to follow-up** **n (%)**	**Followed** **n (%)**	**All** **n (%)**
**Number of Observations**	**153**	**267**	**420**

**Study Area**:			
Qayoomabad	92 (60.1)	173 (64.8)	265 (63.1)
Manzoor Colony	61 (39.9)	94 (35.2)	155 (36.9)

**Mother's Age Group**:			
< 25 years	71 (46.4)	94 (35.2)	165 (39.3)
25-29 years	50 (32.7)	103 (38.6)	153 (36.4)
30-34 years	19 (12.4)	49 (18.4)	68 (16.2)
35 years & above	13 (8.5)	21 (7.9)	34 (8.1)
*Mean age (in years) of mothers (SD)*	*25.45 (4.66)*	*26.46 (4.82)*	*26.09 (4.78)*

**Education**:			
Illiterate	48 (31.4)	63 (23.6)	111 (26.4)
Can read & write	9 (5.9)	14 (5.2)	23 (5.5)
Schooling 1-9 years	53 (34.6)	91 (34.1)	144 (34.3)
10 years of schooling	30 (19.6)	58 (21.7)	88 (21.0)
Schooling 11 years & above	13 (8.5)	41 (15.4)	54 (12.9)

**Working women**:			
Yes	10 (6.5)	21 (7.9)	31 (7.4)
No	143 (93.5)	246 (92.1)	389 (92.6)

**Education of husband**:			
Illiterate	34 (22.2)	40 (15.0)	74 (17.6)
Can read & write	7 (4.6)	11 (4.1)	18 (4.3)
Schooling 1-9 years	41 (26.8)	80 (30.0)	121 (28.8)
10 years of schooling	36 (23.5)	76 (28.5)	112 (26.7)
Schooling 11 years & above	35 (22.9)	60 (22.5)	95 (22.6)

**Mother Tongue**:			
Urdu	40 (26.3)	63 (23.6)	103 (24.6)
Punjabi	40 (26.3)	65 (24.3)	105 (25.1)
Pashto	16 (10.5)	25 (9.4)	41 (9.8)
Hindko	40 (26.3)	92 (34.5)	132 (31.5)
Others**	16 (10.5)	22 (8.2)	38 (9.1)

**Migrant Status**:			
Locals	45 (29.4)	69 (25.8)	114 (27.1)
Immigrant	108 (70.6)	198 (74.2)	306 (72.9)

Ownership of the house*:			
Owned	76 (49.7)	168 (62.2)	242 (57.6)
Did not own	77 (50.3)	101 (37.8)	178 (42.4)

**Persons living in the household**:			
Upto 2	8 (5.2)	15 (5.6)	23 (5.5)
3 to 4	45 (29.4)	57 (21.3)	102 (24.3)
5 to 6	34 (22.2)	62 (23.2)	96 (22.9)
7 & above	66 (43.1)	133 (49.8)	199 (47.4)
*Mean number of persons lived in the household (SD)*	*6.79 (3.98)*	*7.35 (3.93)*	*7.15 (3.95)*

** Includes Saraiki, Sindhi, Balochi, Kashmiri etc.

* p-value < 0.05

**Table 2:** Personal Characteristics of Women in the study area (n = 420)

**Characteristics**	**Lost to follow-up** **n (%)**	**Followed** **n (%)**	**All** **n (%)**
**Number of Observations**	**153**	**267**	**420**

**Total number of pregnancies**:			
Primigravida	34 (22.2)	54 (20.2)	88 (21.0)
Para 1-2	59 (38.6)	105 (39.3)	164 (39.0)
Para 3-4	35 (22.9)	60 (22.5)	95 (22.6)
Para 5 & above	25 (16.3)	48 (18.0)	73 (17.4)
*Mean number of pregnancies (SD)*	*3.44 (2.42)*	*3.52 (2.38)*	*3.09 (0.57)*

**Past history of child death**:			
No	131 (85.6)	233 (87.3)	364 (86.7)
Yes	22 (14.4)	34 (12.7)	56 (13.3)

**Unplanned current pregnancy**.			
Yes	131 (85.6)	218 (81.6)	349 (83.1)
No	22 (14.4)	49 (18.4)	71 (16.9)

**Satisfied with the current life**:			
Yes	149 (97.4)	258 (96.6)	407 (96.9)
No	4 (2.6)	9 (3.4)	13 (3.1)

**Domestic Violence**:			
No	145 (94.8)	241 (90.3)	386 (91.9)
Yes	8 (5.2)	26 (9.7)	34 (8.1)

**Birth Attendant**:			
Doctor	95 (62.1)	161 (60.3)	256 (61.0)
Midwife/LHV	28 (18.3)	54 (20.2)	82 (19.5)
Dai/Neighbour/Relative	30 (19.6)	52 (19.5)	82 (19.5)

**Place of Delivery**:			
Home	41 (26.8)	61 (22.8)	102 (24.3)
Hospital	81 (52.9)	157 (58.8)	238 (56.7)
Maternity Home	31 (20.3)	49 (18.4)	80 (19.0)

**Any complication during delivery**:			.
No	109 (71.2)	171 (64.0)	280 (66.7)
Yes	44 (28.8)	96 (36.0)	140 (33.3)

**Any difficulty soon after birth for the baby**:			
No	95 (62.1)	182 (68.2)	277 (66.0)
Yes	58 (37.9)	85 (31.8)	143 (34.0)

**Any difficulty in breast feeding at birth**:			
No	128 (83.7)	233 (87.3)	361 (86.0)
Yes due to mother	13 (8.5)	23 (8.6)	36 (8.6)
Yes due to baby	12 (7.8)	11 (4.1)	23 (5.5)

No significant association was observed between lost to follow rates in the two study areas. It was 39.4% in Manzoor Colony and 34.7% in Qayoomabad (p-value = 0.341).

Of the total of 267 women who were followed for one year 64.8% were from Qayoomabad and the rest were from Manzoor colony. The overall prevalence of anxiety and depression within one year of childbirth was 28.8 percent (77 women; 95% C.I.: 23.4 to 34.2). Women found to be anxious and depressed at least once included 65 women who had reported only once, 11 women twice and 1 woman thrice (total 90 times). Point prevalence's at 1 month, 2 months, 6 months and 1 year were 5.2 (14/267), 5.2 (14/267), 10.1 (27/267) and 13.1 (35/267) percents respectively.

Majority of the study participants were Muslims (88.4%) and no significant difference were observed in anxiety and depression in Muslims and followers of other religions (p-value = 0.692). Most of the mothers were housewives (92.1%). About one third of the women had a formal education up to schooling of 10 years or more, and a quarter of them had never been to school. About two third of them reported ownership of homes; this was more in Manzoor Colony (73.4%) as compared to Qayoomabad (56.1%).

Mother tongue was used as a surrogate marker for immigration, Malabari, Sindhi and Balochi are the languages of the original inhabitants of the coastal areas of Karachi and Urdu is spoken by the people who migrated to Karachi at the time of partition of India in 1947 i.e. 60 years back; for the purpose of analysis they were grouped together as locals. As Karachi is a large business hub; workers from other areas of Pakistan come and go in search of jobs and they speak Punjabi/Hindko/Pushto, mothers speaking these languages were considered immigrants. In Qayoomabad majority of the mothers were immigrants (89.0%) while in Manzoor Colony only 46.8% were immigrants. A higher prevalence of anxiety and depression was observed among immigrants, however it was not statistically significant (p-value = 0.558). Majority of the women (96.6%) in both areas were satisfied by their current life (p-value = 0.765). Similarly a large number of women (81.6%) had unplanned current pregnancy (p-value = 0.017). A small number of women (5.6%) reported verbal or physical abuse by a family member for themselves or their children (p-value = 0.132).

More than two thirds of the childbirths were attended by a trained person such as a doctor (60.3%) or a midwife/Lady Health Visitor (20.2%) and were conducted in either a hospital (58.8%) or a maternity home (18.4%); in spite of this about one third of the mothers reported complications during or after childbirth.

Distribution of anxiety and depression for different characteristics of mothers are given in table 3. Multivariable analysis is provided in table 4.

**Table 3:** Univariate analysis of mothers and children characteristics (n = 267)

**Characteristics**	**Total Participants**	**Overall Prevalence ** **of PPAD%**	**Odds Ratio (95% C.I.)**
**Study Area**:			
Qayoomabad	173	28.3	1
Manzoor Colony	94	29.8	1.07 (0.62, 1.86)

**Mother's Age Group**:			
< 25 years	94	24.5	1
25-29 years	103	28.2	1.21 (0.64, 2.29)
30-34 years	49	34.7	1.64 (0.77, 3.48)
35 years & above	21	38.1	1.90 (0.70, 5.16)

**Working women**:			
Yes	21	19	1
No	246	29.7	1.79 (0.58, 5.51)

**Migrant Status**:			
locals	69	26.1	1
Migrants	198	29.8	1.20 (0.65, 2.23)

**Past history of child death**:			
No	233	27.5	1
Yes	34	38.2	1.64 (0.77, 3.46)

**Unplanned current pregnancy**			
Yes	218	25.7	1
No	49	42.9	2.17 (1.14, 4.12)

**Domestic Violence**:			
No	241	26.6	1
Yes	26	50	2.77 (1.22, 6.28)

**Any complication during delivery**:			
No	171	27.5	1
Yes	96	31.3	1.20 (0.69, 2.07)

**Any difficulty soon after birth for the baby**:			
No			
Yes	182	24.7	1
	85	37.6	1.84 (1.06, 3.20)

**Any difficulty in breast feeding at birth**:			
No	233	26.6	1
Difficulty due to mother	23	43.5	2.12 (0.88, 5.08)
Difficulty due to child	11	45.5	2.30 (0.68, 7.80)

**Table 4:** Multivariable analysis of mothers and children characteristics (n = 267)

**Characteristics**	**Adjusted Odds Ratio (95% C.I.)**
**Study Area**:	
Qayoomabad	1
Manzoor Colony	1.31 (0.72, 2.38)

**Mother's Age Group**:	
< 25 years	1
25-29 years	1.31 (0.67, 2.56)
30-34 years	1.27 (0.57, 2.86)
35 years & above	1.82 (0.63, 5.24)

**Unplanned current pregnancy**:	
Yes	1
No	2.11 (1.04, 4.29)

**Domestic Violence**:	
No	1
Yes	2.82 (1.15, 6.89)

**Any difficulty in breast feeding at birth**:	
No	1
Difficulty due to mother	2.98 (1.20, 7.43)
Difficulty due to child	3.13 (0.88, 11.13)

In the univariate analysis, unplanned current pregnancy (p-value = 0.017), domestic violence (verbal or physical abuse by any family member towards the mother/children) (p-value = 0.012), and child having any difficulty soon after birth (cyanosis/apnea/flaccidity) (p-value = 0.03) were found to be significantly related to postpartum anxiety and depression. Other important characteristic was having difficulty in breast feeding the newborn (p-value = 0.125). Study area (p-value = 0.801), which was used as proxy for immigrant status and mother's age group (p-value = 0.459) was considered biologically important variable for inclusion in multivariable analysis. No significant difference was observed for parent's educational status, mother's working status, household income, ownership of the household and person living in the household among women with postpartum anxiety and depression and other women.

In multivariable analysis, the variables included in the model were study area, age of women, unplanned current pregnancy (p-value = 0.038), domestic violence (verbal or physical abuse towards mother or children by any family member) (p-value = 0.023), and having difficulty in breast feeding the newborn (p-value = 0.021).

## Discussion

In this study an overall prevalence of postpartum anxiety and depression was found to be 28.8% and point prevalence's at 1 month, 2 months, 6 months and 12 months were 5.2, 5.2, 10.1 and 13.1 percents respectively. Whereas studies conducted in Pakistan by Rahman et al, which were done in rural communities had point prevalence's of 28 and 36 percents respectively [11,14]. In another study, he reported persistent postpartum depression of 56 percent [15]. Other studies have reported point prevalence's of 24% - 42% in urban tertiary care settings in Pakistan [8,13]. The reasons for low point prevalence's in our study might be that this was an urban community based study and counselling was provided to the anxious/depressed women.

In our study, an association of postpartum anxiety and depression with domestic abuse, resentment about current pregnancy and difficulty in breast feeding was found. Several other studies have also found higher rates of depression in women suffering from domestic abuse [28-32]. Unwanted pregnancy is also been reported as an increased risk for PPD [33]. In one study conducted in a tertiary care hospital, Karachi; young age and low level of education in mothers had emerged as significant predictors [8] where as in our study these factors were not found to be significant in multivariate analysis. No significant association with employment status of mothers, family structure, and sex of the baby was found in our study which is similar to one of the urban tertiary care setting study in Pakistan [8]. But studies from South Asia have reported that giving birth to a female child is also associated with PPD [9] especially in mothers having more than two female children [11,34]. In South Asian culture there is a preference for male child and mothers are usually blamed for giving birth to a female child. One study conducted by Rahman and Creed in a rural sub-district of Pakistan has reported poverty, having 5 or more children, uneducated husband and lack of confidant or friend are significantly associated with persistent depression [15]. A literature review by Klainin and Arthur on postpartum depression in Asian culture has also reported poverty, unwanted pregnancy, preference of infant's gender to be associated with PPD [35].

Many studies have reported an association of financial difficulties with PPD, [18,36] but in our study no such association was found which is similar to the findings reported by Josefesson et al [37].

Depressive symptoms during pregnancy have been consistently identified as the single strongest risk factor for postpartum depression[20,38]. Unfortunately in our study it was not possible to obtain reliable information regarding occurrence of prior anxiety/depression as these are not considered as medical problems in these communities. Medical help is seldom sought and even when it is done, depression is poorly diagnosed and euphemisms are used even by the doctors because of the stigma associated with the diagnosis. As depression is either attributed to supernatural causes or is taken as a shameful weakness of mind.

The association with difficulties in breast feeding at birth should be further explored by future studies. In our study, difficulty in breastfeeding at birth did not seem to be directly related with postpartum anxiety and depression as point prevalence's in the initial assessments were lower than the later assessments.

A relationship between stressful life events and postpartum depression was found in a meta-analysis of 15 studies on life events [20]. The results of our study are similar, as the significant factors that emerged were all stressful life events i.e. domestic abuse, undesired current pregnancy and difficulties in breast feeding.

Our study had some limitations; firstly, the unavailability of characteristics of 231/651 women, who did not consent for participation in the study for comparison. Secondly a large lost to follow rate among consenting women due to frequent change in residence and refusals after providing initial consent because of stigma associated with mental illness. Third, we had only twelve women who were anxious and depressed more than once; out of those nine women were found positive on two consecutive occasions and not on all four occasions. Therefore, the assessment of risk factors for persistent affective disturbance could not be determined. Fourth, we did not assess the past history of anxiety/depression particularly during current pregnancy hence are unable to comment that infants of mothers with postpartum anxiety/depression are more vulnerable rather than the conditions themselves existing independently and exerting adverse effects on infants. Another limitation of the study population comprised of only low to lower middle socioeconomic classes.

## Conclusion

Domestic abuse has to addressed through women's empowerment by a socio cultural change which does not fall directly under the preview of medical practitioners, but unwanted pregnancy is not entirely a result of patriarchy several factors directly related to our profession are involved like, lack of knowledge of contraceptive methods, accessibility and cost of contraceptives which can be changed through public health measures. An incidental finding is that more than two third of the deliveries were attended by a trained person such as a doctor (60.3%) or a midwife/Lady Health Visitor (20.2%) and were conducted in either a hospital (58.8%) or a maternity home (18.4%), and despite this, about one third of the deliveries had complications; and stressful events during puerperium are a risk for PPD and prevention of postpartum complications could contribute to reducing prevalence of PPD. More over clinicians need to be aware of the burden of PPD, and its grave consequences, and would do well to make screening for anxiety and depression a part of routine post-natal care.

## Competing interests

The authors declare that they have no competing interests.

## Authors' contributions

NSA conceived and designed the study and prepared the manuscript. BSA designed the study questionnaire and provided intellectual feedback. ISA managed, analyzed and interpreted the data. All authors read and approved the final manuscript.

## Pre-publication history

The pre-publication history for this paper can be accessed here:


